# Fentanyl Research: Key to Fighting the Opioid Crisis

**DOI:** 10.3390/jcm14155187

**Published:** 2025-07-22

**Authors:** Cristina Rius, Antonio Eleazar Serrano-López, Rut Lucas-Domínguez, Andrés Pandiella-Dominique, Carlos García-Zorita, Juan Carlos Valderrama-Zurián

**Affiliations:** 1 UISYS Group, Department of History of Science and Information Science, Faculty of Medicine and Dentistry, University of Valencia, 46010 Valencia, Spain; 2 Unit Associated with the Interuniversity Institute for Advanced Research on the Evaluation of Science and the University (INAECU), UC3M-UAM, 28903 Madrid, Spain; 3 Spanish National Centre for Cardiovascular Research (CNIC), 28029 Madrid, Spain; 4 Centro de Investigación Biomédica en Red de Enfermedades Cardiovasculares (CIBERCV), 28029 Madrid, Spain; 5 Laboratorio de Estudios Métricos de Información (LEMI), Departamento de Biblioteconomía y Documentación, Universidad Carlos III de Madrid, 37008 Madrid, Spain; 6 Instituto Interuniversitario de Investigación Avanzada sobre Evaluación de la Ciencia y la Universidad (INAECU), Universidad Carlos III de Madrid, Universidad Autónoma de Madrid, 28049 Madrid, Spain; 7 Centro de Investigación Biomédica en Red de Cáncer (CIBERONC), 28029 Valencia, Spain

**Keywords:** fentanyl, health policy, scientific research, health care information, health care managers, opioids crisis, opioids mortality waves

## Abstract

**Background/Objective:** Fentanyl plays a pivotal role in the opioid epidemic, defined by four waves of overdose deaths. To analyse fentanyl research trends, examining its links to mental health, pharmaceutical development, healthcare, diseases, and pathophysiology within the broader social and health context of the time. **Methods:** To understand the evolution of scientific publications on fentanyl and its relationship to the opioid crisis, a search using Web of Science Core Collection and PubMed was conducted. A total of 53,670 documents were retrieved related to opioid scientific production, among which 1423 articles (3%) focused specifically on fentanyl. The 21,546 MeSH terms identified in these documents were analysed by publication year and specific fields: Psychiatry and Psychology, Chemicals and Drugs, Healthcare, Diseases, and Phenomena and Processes. R-statistical/FactoMineR libraries were used for the correspondence analysis. **Results:** In the first overdose death wave, research focused on improving therapies and reducing side effects. The second wave emphasised detoxification methods with naltrexone, methadone, and behavioural therapies. The third wave addressed psychological treatments and HIV-syringe-sharing prevention. The fourth wave prioritised less addictive analogues and understanding consumer profiles to combat the epidemic. **Conclusions:** Fentanyl research has evolved alongside real-world challenges, reinforcing the connection between patients’ needs, healthcare professionals’ roles, illicit users, policymakers, and the research community’s contributions to addressing both therapeutic use and its broader societal impact. These findings highlight the necessity for an interdisciplinary approach to scientific research integrating prevention, treatment, education, legal reform, and social support, emphasising the need for public health policies and collaborative research to mitigate its impact.

## 1. Introduction

The prescription opioid epidemic in the United States (U.S.) is one of the most challenging public health crises in recent history. The crisis, which began in the 1990s, was triggered when opioids were increasingly prescribed for pain, with little regard for their addictive potential and the risks associated with their use. Pharmaceutical companies actively promoted the prescription of these painkillers [1], touting their safety and efficacy, which led to mass consumption and, over time, misuse, triggering dependence and addiction, and resulting in high rates of overdose and death [2]. Prescription opioids, such as oxycodone and hydrocodone, became the gateway to the abuse of more potent substances, including illicit fentanyl and heroin. The crisis was exacerbated by over-prescribing, lack of education on pain management, and inadequate regulation and monitoring of prescribing [3].

In 2018, one in five adults in the U.S. had an opioid prescription, with higher rates observed among women across all age groups [4]. Oxycodone (short-acting) was the second most commonly prescribed opioid, accounting for 23.6% of prescriptions, behind hydrocodone. Meanwhile, oxycodone (long-acting) and fentanyl accounted for just 1.1% of total opioid prescription [5]. Transdermal fentanyl initiation was primarily prescribed to women and individuals aged 65 years and over who had previously been treated with oxycodone [6]. Demographic studies show that opioids are prescribed more frequently to white people for acute and chronic pain. Studies of opioid prescribing in emergency departments, including the prescribing of hydrocodone, oxycodone, fentanyl, and others, but not codeine, revealed that Black people, as well as people of other ethnicities (including Hispanic and Asian groups), were less likely to be prescribed opioids for almost all types of pain [7,8,9]. Similarly, studies of prescription opioids have reported that adults from higher socioeconomic backgrounds, as measured by educational attainment and household income, were less likely to have used these drugs than those from lower socioeconomic backgrounds [10].

Since 1990, successive waves of opioid-related mortality have occurred for different reasons. Initially, uncontrolled prescribing led to overdoses of opioid analgesics; the second wave was characterised by an increase in heroin-related deaths; the third wave was driven by synthetic opioids [11]; and the fourth wave by the use of fentanyl in combination with other psychostimulant drugs [12].

In particular, different patterns of illicit drug utilisation have been observed over the past decade, with cocaine overdoses remaining stable and psychostimulant overdoses increasing [13]. The mortality rate associated with illicit drug use in the U.S. before 2010 was increased by psychostimulant use even without opioid consumption, but it was not until 2015 when opioid with cocaine or psychostimulant intake emerged, raising the number of opioid-associated deaths, with significant differences observed in the geographic distribution of overdose cases and the habits of opioid use along with other illicit drugs across the U.S. [14]. Moreover, differences have also been noted in the profile of opioid consumers alone or in combination [13]. Fentanyl is an opioid drug developed and widely used as a potent analgesic, but in the second decade of the 21st century it entered the illicit market in the U.S. [15]; however, the reasons and specific mechanism by which it escaped non-therapeutic use remain unclear, and it has become a critical public health and safety issue [16].

Currently, the U.S. is suffering a “fourth wave” of overdose mortality dominated by the use of psychostimulants (methamphetamine) and cocaine, which, in combination with opioids, mainly fentanyl, complicates overdoses and positions this opioid as the main mortality factor [17]. The recent escalation in stimulant deaths overlaps with the widely existing opioid epidemic, suggesting a lethal interaction between the use of different substances with fentanyl [18]. Furthermore, the rising tide is intensified by multiple factors, including the accessibility of fentanyl and diminishing therapeutic options, driving patients towards self-use [11]. This pattern highlights the increasing complexity of the opioid overdose crisis (OOC), whether due to opioid use alone or in combination with cocaine or methamphetamine, and the need for multifaceted intervention strategies. In addition, this situation extends geographically to other regions like Australia, New Zealand, and Europe; while the U.S. leads the ranking of global fentanyl use in 2021 (prescribed and illicit), Germany, Spain, and Italy are close behind (19.3%, 14.5%, 11.8%, and 6.3%, respectively) [19]. In the United Kingdom, opioid prescribing rates have now reached levels comparable to those in the U.S., and despite a recent decline, the frequency of opioid prescribing remains high [20]. Although prescriptions have risen in most European countries, opioid-related issues like hospitalisations, dependence, or overdose deaths have not significantly increased, unlike the U.S. crisis. Scotland is the notable exception, with opioid overdose mortality comparable to that of the U.S., diverging worryingly from Europe [21]. To curb opioid abuse and misuse, laws and stringent regulations have been introduced. Nevertheless, these measures linked to a reduction in consumption based on pharmacological prescription have negatively affected the availability of opioids for medical purposes [22].

Previous investigations show that between 1995 and 2016, opioid research was dominated by studies in the fields of neurology and analgesia, with predominantly clinical trials on oxycodone in relation to neuropathic pain [23]. However, the research on addiction and consumption of opioids was less represented despite the high mortality rates and their importance worldwide. In the same line, during this period, the most studied opioids were endogenous opioids (endorphins, enkephalins, and dynorphins), which reflects a great interest in the scientific community to know the mechanism of action of opioids in the body (receptors, physiopathological production dynamics, etc.) [24]. In contrast, few studies have examined the landscape of scientific production on fentanyl in recent years, but it is of great interest to identify the main research topics and their relationship with trends in opioid use, misuse, and mortality that characterise the current public health crisis; this knowledge helps to predict the priority lines of research, the needs, and the areas for improvement that serve as a guide for the development of regulatory, epidemiological and public health policies in different geographical areas.

In the face of this OOC, the aim of this study was to analyse the evolution and trends of fentanyl research and to assess its relationship with the social and health situation at the time, looking at it from different perspectives: mental health, pharmaceutical development and use, health care, diseases, and related pathophysiological phenomena.

## 2. Methodology

In line with the stated objective, an analysis of annual data on international scientific research on opioids up to 2022 was carried out using the Web of Science Core collection database (WoS).

To this end, a search equation was established to retrieve all articles and reviews published on opioids, excluding papers classified in the WoS in the Meso Citation Topics with non-relevant items (Supplementary File S1), and a total of 53,670 documents were retrieved. Of these, those documents containing a PubMed database identifier (PMID) were selected (47,442 records) and cross-referenced with the PubMed database, eliminating papers that did not have Medical Subject Headings (MeSH) terms assigned to them. The final opioids database resulted in 47,405 documents, which contained a total of 1,073,156 MeSH terms (10,867 unique terms).

Subsequently, the opioids database was stored in an SQL database management system (MariaDB), which allows queries to be made directly on any field, including MeSH terms, and this filter was used to obtain the scientific production on Fentanyl used below, corresponding to a total of 1423 publications (representing 3% of the total scientific production of opioids), which in turn contain 21,546 MeSH terms (2006 unique terms). A similar method was used in previous studies [25,26].

**Figure 1:**
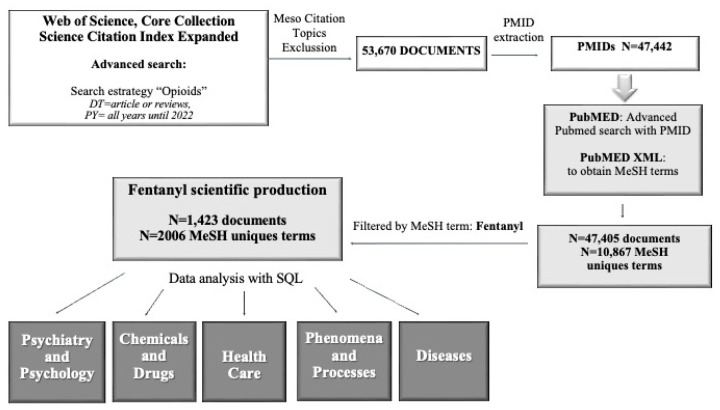
Flow diagram of Fentanyl database search strategy.

For the analysis of fentanyl documents, the publication date of the records and their associated MeSH terms were evaluated using the R statistical package [27] combined with the FactoMineR library [28], to calculate and graph the correspondence analyses presented in the Results Section. Correspondence analysis is a multivariate technique that allows us to examine the relationships between variables in a contingency table. The versatility of this data visualisation method, which can be applied to a wide variety of situations, has been highlighted by Greenacre (2007) [29]. In the present study, this statistical method allows us to identify and visualise the relationships between the MeSH terms for each of the MeSH branches considered—Psychiatry and Psychology (Branch F), Chemicals and Drugs (Branch D), Health Care (Branch N), Diseases (Branch C), and Phenomena and Processes (Branch G) (Figure 1)—and the publication date of the articles they describe, facilitating the graphical representation by means of a biplot, where the proximity or distance of the points reflects the degree of relationship between the categories represented in the rows and columns of the variable matrix. The aim of these graphs is to show the thematic evolution of fentanyl research over time from different perspectives. The origin of the coordinates is set in such a way that the distances between descriptors and years of publication are balanced, so that, as usual in the Cartesian plane, four quadrants characterise the thematic interest of the publications. This analysis allows us not only to identify clusters, but also to understand the distribution of documents according to MeSH terms and their publication dates.

Due to the volume of terms retrieved from each of these MeSH branches, publication thresholds were set to determine the number of terms to be displayed and examined in each graph. These thresholds were set between 20 and 5, that is, terms appearing in 20 or more publications (Branch D) or those appearing in 5 or more publications (Branch C), respectively.

## 3. Results

### 3.1. Research on Fentanyl in the Field of Psychiatry and Psychology

**Figure 2:**
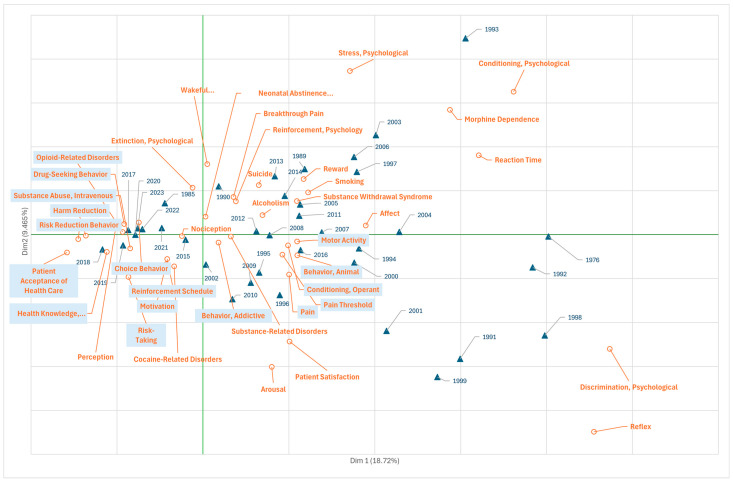
Scientific production on fentanyl (1972–2022) represented by the MeSH terms from Branch F—“Psychiatry and Psychology” (correspondence analysis, total variability, 28.2%). Frequency threshold of the terms, 5.

The most studied aspects in scientific publications on fentanyl related to psychology over time are shown in Figure 2. As in the rest of the graphs, the correspondence analysis in the two dimensions with the highest explanatory variability (Dim. 1 = 18.7%, Dim. 2 = 9.5%) represents the joint space of MeSH descriptors and years of publication.

Thus, in the lower right quadrant (fourth quadrant) of Figure 2, publications between 1996 and 2010 (the period coinciding with the first wave) are observed, with a thematic predominance of documents using terms such as “pain”, “pain threshold”, “animal behaviour”, and “motor activity”. This behaviour shows the interest of researchers in characterising in detail the properties of fentanyl in pain management from basic research by studying animal models, as well as the mechanisms of learning and behaviour modification (“conditioning operant”) related to addictive behaviour (“behaviour addictive”) induced by fentanyl during the same period.

The third quadrant, represented by publications from 2015 to 2020, coinciding with the third wave phase, tends to analyse psychological mechanisms responsible for dependence and addiction in order to implement different reinforcement strategies to reduce habituation. On one hand, there is a predominance of the topics “reinforcement schedule” and “patient acceptance of healthcare motivation” and tools to combat drug-seeking behaviour and motivation (“drug-seeking behaviour”, “choice behaviour”, and “health knowledge, attitudes and practice”), and, on the other hand, there is a tendency towards topics related to the reduction in the harm caused by addiction and overdose to consumers and their environment (“harm reduction”, “risk taking”, “risk-reduction behavior”, and “opioid-related disorders”).

This period also includes publications on intravenous fentanyl abuse (“substance abuse, intravenous”), reflecting not only a focus on the specific medical problems and public health consequences, but also on the broader sociological dimensions of the crisis, such as structural inequality, stigma, and the marginalisation of affected communities, which have exacerbated the impact of the OOC.

### 3.2. Research on Fentanyl in the Field of Chemicals and Drugs

**Figure 3:**
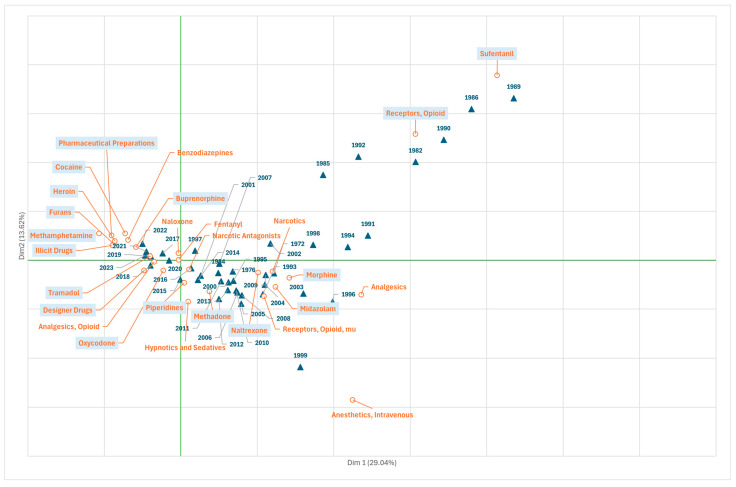
Scientific production on fentanyl (1972–2022) shown by MeSH terms from Domain D Chemicals and Drugs” (correspondence analysis, total variability, 42.6%). Frequency threshold of the terms, 20.

Figure 3 shows the biplot for Branch D, highlighting the focus on drugs and chemical and pharmacological development of fentanyl research. From the beginning of the first wave (the 1980s and 1990s) to the end of the first quadrant, research is focused on pharmacological studies to characterise specific opioid receptors (“receptor opioid”) and to advance knowledge of the structure, mechanisms of action, and pharmacological effects of various opioid derivatives, such as “sulfentanil”.

In the fourth quadrant (Figure 3), which encompasses the final years of the 1990s and extends through the 2000s, including some years of the first decade of the 2000s, when the first wave of the OOC spread, publications were redirected towards the study of other pharmacological groups used in parallel with or as an alternative to fentanyl in clinical practice in the field of analgesia and anaesthesia, such as benzodiazepines (“midazolam”), and, simultaneously, in lines of research focusing on other opioid derivatives, highlighting “morphine”, “methadone”, and the opioid antagonist “naltrexone”, suggesting a focus on the development of treatments for opioid dependence, detoxification, and overdose, which reflects a shift towards clinical application and management of the adverse effects generated by opioid dependence.

In recent years, at the end of the third wave and the beginning of the fourth wave, the trend that centred on the development of new molecules for therapeutic use derived from “piperidines” and “furans” has transitioned to investigation of other substances for recreational or illicit use, identifying a cluster of terms (second and third quadrants of Figure 3) that establishes four priority lines of research: (i) the development of new drugs derived from opioids, constituted by the terms “designer drugs”, showing an interest in new substances and the impact of these designer drugs on society; (ii) the problem derived from the consumption of fentanyl with other illicit drugs in combination, made up of the terms “cocaine”, “illicit drugs”, “heroin”, or “methamphetamine”; (iii) the strategies to manage and combat fentanyl dependence and overdose, consisting of the terms “buprenorphine” and “pharmaceutical preparations”, reflecting the emerging concern about the problem caused by the increase in the use of synthetic drugs combined with fentanyl on the market; and (iv) the adequate prescription of “oxycodone” or “tramadol” for the treatment of pain.

### 3.3. Research on Fentanyl in the Field of Health Care

**Figure 4:**
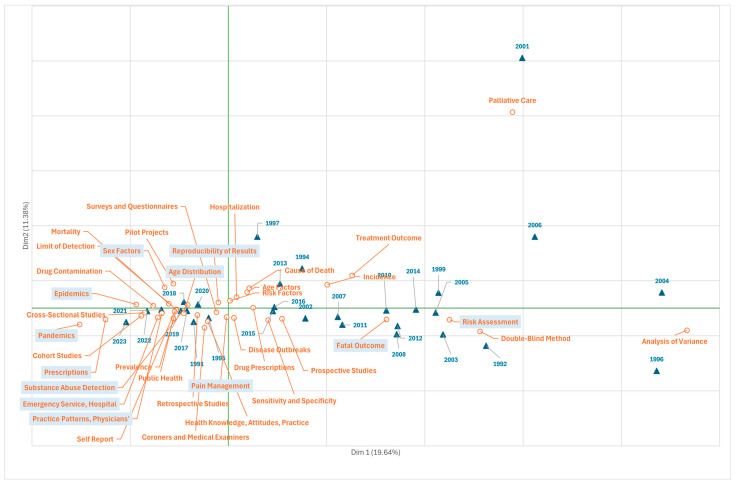
Scientific production on fentanyl (1972–2022) presented by the MeSH terms from Branch N “Health care” (correspondence analysis, total variability, 31%). Frequency threshold of the terms, 10.

The health care aspects of fentanyl research evaluated by correspondence analysis produce a biplot in which the first dimension distinguishes between papers published from the 1990s to some years in the first decade of the 2000s (on the right of the *x*-axis), when there was more interest in “risk assessment” and the fatal consequences (“fatal outcome “) of fentanyl abuse and overdose (Figure 4) coinciding with the rise in prescription opioid overdose deaths started in 1990 (first wave).

In the most recent period (2017–2023, fourth wave), to the left of the *x*-axis, topics are related to health services studies analysing sexual factors and the age of those who use fentanyl (“sex factors”, “age distribution”), prescriptions for pain management (“prescriptions”, “pain management”), systems for detecting drug abuse (“substance abuse detection”), and medical practice patterns (“practice patterns physicians”) in hospital emergency departments (“emergency service hospital”) and other centres involving patients who use fentanyl. Also highlighted over this period are epidemiological retrospective studies, cohort studies, cross-sectional prevalence studies, surveys and questionnaires of patients and their communities, and pilot projects, with particular attention to the reproducibility of results (“reproducibility of results”). Finally, it should be noted that in recent years terms such as “pandemics” and “epidemics” have become relevant.

### 3.4. Research on Fentanyl in the Field of Diseases

**Figure 5:**
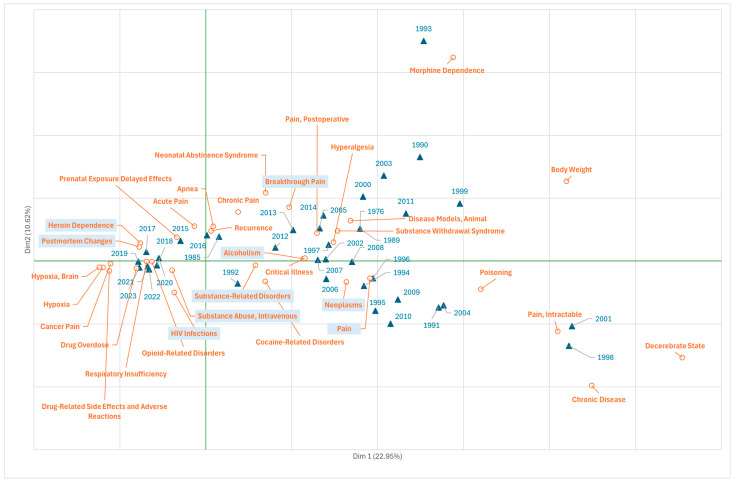
Scientific production on fentanyl (1972–2022) exhibited by the MeSH terms from Branch C “Diseases” (correspondence analysis, total variability, 33.6%). Frequency threshold of the terms, 5.

The study of the terms in Branch C (Diseases) is presented in Figure 5, which shows through the X-axis (23% variability) two distinguished time periods; the first (on the right of the *x*-axis) includes publications up to the middle of the second decade of the 2000s, which focus on the use of fentanyl in the modulation of oncological pain (“neoplasms”, “pain”), post-operative pain, and breakthrough pain (“breakthrough pain”), derived from critical illness or hyperalgesia, and the addictive consequences derived from treatment with fentanyl, including alcoholism (“substance-related disorders”, “alcoholism).

In a second period (on the left of the *x*-axis), from the middle of the second decade onwards, publications focus on the adverse effects of drug dependence and overdose, comprising cerebral hypoxia or respiratory failure, and on forensic research (“postmortem changes”). Also of concern are the abuse of intravenous substances such as heroin (“heroin dependence”, “intravenous drug abuse”) and HIV infection (“HIV infection”), coinciding with the rise in heroin overdose deaths (second wave).

### 3.5. Research on Fentanyl in the Field of Phenomena and Processes

**Figure 6:**
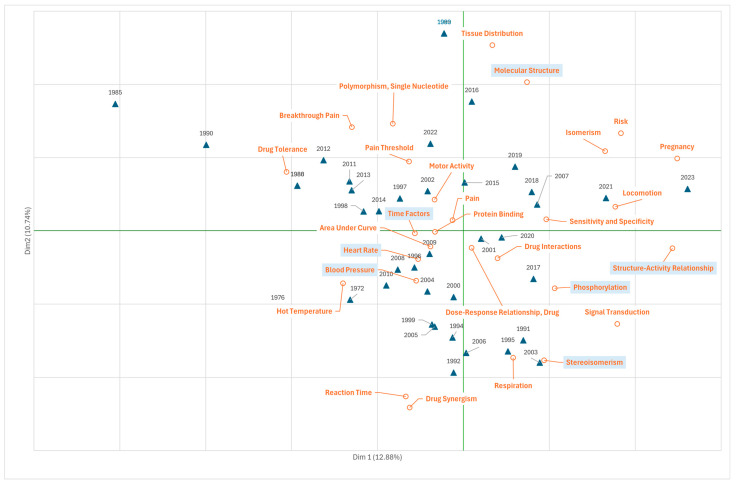
Scientific production on fentanyl (1972–2022) according to MeSH terms from Branch G “Phenomena and processes” (correspondence analysis, total variability, 23.6%). Frequency threshold of the terms, 7.

Figure 6 illustrates the biological, chemical, physical processes, and phenomena studied in publications on fentanyl. As in the previous biplot, the analysis carried out makes it possible to differentiate two trends in the publications along the *x*-axis (12.9% variability), which marks a cluster (to the left of the *x*-axis) covering most of the period studied and focusing on pathophysiological processes related to fentanyl use, the dose–response relationship, time factors in the response (“time factors), mechanisms of drug tolerance or effects on the cardiovascular system (“heart rate”, “blood pressure”) and motor activity, and pain threshold. The second cluster (to the right of the *x*-axis) is made up of publications from the middle of the second decade and relates to the molecular structure of substances (“molecular structure”, “structure-activity relationship”, “phosphorilation”, “stereosimerism”, “structure-activity relationship”) and the development of studies on the sensitivity and specificity of physiological responses to fentanyl or on the interaction of substances consumed, corresponding with the emergence of the use of fentanyl with stimulants (wave 4).

## 4. Discussion

In recent decades, the scientific community has witnessed several waves of opioid use, misuse, and dependence, each characterised by different patterns of use/consumption, different substances, and different social and health consequences [11,30]. Fentanyl is an important therapeutic drug, 50–100 times more potent than morphine, which is widely used in surgical practice and pain relief. However, oxycodone (the second most widely consumed opioid after fentanyl) and morphine are used in clinical settings to relieve pain. Both have similar analgesic potency and pharmacokinetics and pharmacodynamics, which are much slower than those of heroin and fentanyl [31,32]. Clinical practice guidelines recommendations currently note a significant increase in the risk of fentanyl dependence when the daily dose exceeds 90 milligrams of morphine equivalents, although some countries have less restrictive guidelines [33]. In all cases, prescribing the highest risk of tolerance and dependence is associated with high doses, overlapping prescriptions of opioids and benzodiazepines, and the use of extended-release/long-acting opioids for acute pain [34].

Recent studies have revealed that the fentanyl transdermal patch form is one of the substances most frequently associated with dependence [35], versus transmucosal formulations [36]. Additionally, misuse of transdermal patches, such as chewing or transmucosal use, is common among people who use drugs [35]. The different forms of intranasal administration do not differ in terms of dependence [37].

The demographic profile of oxycodone use in a clinical setting is adults aged 40–60 and over 60, with slightly higher prevalence among women than men. Additionally, its use is more prevalent in the non-Hispanic white population than in the non-Hispanic black population. Hispanic women are more likely to use opioid analgesics than Hispanic men [38]. In this sense, oxycodone is associated with a more traditional clinical profile involving older, female, and white patients. In contrast, fentanyl, particularly in its illicit form, has emerged with a more complex and lethal pattern, disproportionately affecting young males and marginalised populations. While both opioids have played a key role in the crisis, fentanyl’s immediate lethality requires urgent harm reduction strategies that are tailored to the different demographics affected [32].

The MeSH terms of publications in each of the fields analysed, Psychiatry and Psychology (Branch F), Chemicals and Drugs (Branch D), Health Care (Branch N), Phenomena and Processes (Branch G), and Diseases (Branch C), have responded to these crises by adapting and developing their approaches and methods.

This study presents the published scientific literature on fentanyl and its relationship to these waves from the perspectives of psychology, health care, drug and chemical development, and pathologies and phenomena arising from the OOC.

### 4.1. First Wave: Prescription Opioid Overdose Deaths

The first wave began in the mid-1990s and resulted from health care providers’ difficulty in effectively managing severe pain, coupled with an overreliance on the adverse effects of opioid analgesics, which led to the overprescribing of these drugs [39]. Unfortunately, this approach has led to significant increases in drug misuse, diversion, opioid use disorder, and overdose. In addition, studies such as that of Manchikanti et al. (2012) describe how overprescribing was fuelled by aggressive marketing campaigns and an underestimation of the risks of addiction [30]. During this period, research in psychiatry and psychology focused on understanding the basis of addictive behaviour generated by the misuse of fentanyl alone or in combination with other substances, and on developing interventions to treat addiction. It also coincides with a general interest in characterising the analgesic properties of fentanyl from all angles (physiology, pathological pain) [40].

At the same time, studies in the field of chemicals and drugs during this period focused on the need to increase knowledge of opioid receptors and the pharmacological characterisation of opioid derivatives in order to improve clinical treatments [41], and to optimise therapeutic strategies to better manage the risks associated with the prescribed doses and opioid abuse that characterised the era [42]. This illustrates a continued high level of commitment by the scientific community to improve medical therapies and reduce their adverse effects, with innovations in anaesthetic and analgesic formulations, combined with other non-opioid drugs (‘midazolam’) for medical procedures [43,44]. This period reflects a continuing interest in maximising the clinical use of fentanyl while controlling its addictive properties.

### 4.2. Second Wave: Expansion of Heroin Use and Increase in Overdoses

The second wave was described by an increase in heroin use as a result of restrictions on opioid prescribing [11]. Dickson-Gomez J et al., 2022, explain how individuals dependent on prescription opioids switched to heroin use because it was more accessible and cheaper [45]. Consequently, illicit fentanyl, which was often mixed with other drugs, mainly heroin, increased the risk of overdose due to the difficulty of correct dosing. In parallel, there has been an increased interest in research on the social and health consequences and risk assessment to estimate the lethal effects of injecting heroin together with fentanyl [46], both of which are obtained illegally, and an increase in studies on educational programmes that emphasise the importance of harm reduction strategies through safe syringe use [47].

In this context, research has shifted towards harm reduction and addiction treatment strategies, with the flourishing of “operant conditioning” and “behaviour addictive” studies exploring behavioural therapies using operant conditioning techniques (based on reward and punishment) [48,49], which have been shown to be a powerful psychological tool for modifying addictive responses to opioids [50]. Similarly, there is growing interest in pharmacology to characterise substances such as methadone or naltrexone as more effective and safer methods of detoxification and overdose reversal [51].

### 4.3. Third Wave: Mass Use of Fentanyl and Fentanyl Analogues

The third wave, which began around 2014, is defined by the abuse of fentanyl and its highly potent synthetic analogues alone [52].

From a psychological point of view, the increase in substance use disorders and dependence on highly potent addictive substances that characterises this period is reflected in the surge in research on “drug seeking behaviour”, “reinforcement schedule”, and “risk reduction behaviour”, as this drug rapidly generates psychological dependence when the user begins to associate fentanyl use with relief from stress, anxiety, or depression, creating a cycle of abuse that is difficult to break [53]. In fact, people with mental disorders, particularly thought disorders like schizophrenia or bipolar disorder, are at increased risk of both fatal and non-fatal opioid overdoses [54]. Downs et al. (2025) found that nearly half of emergency patients using illicit fentanyl had psychiatric comorbidities (mood disorders, psychotic disorders, and anxiety disorders) with 21.8% having multiple diagnoses [55].

### 4.4. Wave 4: Massive Use of Fentanyl with Stimulants

Since 2019, the fourth wave has begun and, in contrast to the third wave, the negative effects and the number of overdoses and deaths caused by fentanyl have increased due to its use in combination with other stimulant drugs [11]; this has attracted the interest of researchers in this field (MeSH terms “cocaine”, “illicit drugs”, “heroin”, or “methamphetamine”).

The proliferation of more powerful and difficult-to-detect fentanyl analogues, which pose an even greater risk of fatal overdose due to their even more extreme potency when combined with other drugs such as methamphetamine, cocaine, or heroin, increases the complexity of treatment and the need for more sophisticated medical approaches [56]. In this sense, it is worth noting the efforts of researchers to synthesise new drugs analogous to opioids but capable of reducing their addictive effects without losing analgesic efficacy, such as furan derivatives, are noteworthy [57,58].

On the other hand, similar to the third wave period, researchers continue to show a strong interest in the sharing of syringes for intravenous use of this drug and, subsequently, in HIV infections among people who use fentanyl [45].

The rise in overdose mortality due to fentanyl has led to a significant increase in the distribution of naloxone and other opioid antagonists to rapidly reverse the effects of overdose (“buprenorphine”, “pharmaceutical preparations”) [59]. Research in this period highlights the value of buprenorphine to alleviate refractory depressive episodes [60,61], complemented from a medical and public health point of view with educational programmes related to buprenorphine/naloxone and the risks derived from the OOC [62]. From a psychological perspective, there has been a growing interest in unpacking strategies for the detoxification and rehabilitation of patients addicted to the use of fentanyl (MeSH term “drug seeking behaviour”).

The OOC deaths attributed to illicit fentanyl since 2013 are unprecedented, as fentanyl production has emerged as a positive supply shock, increasing its use relative to other opioids (heroin) [18], causing major social and health concerns, and making it the subject of intense research in several fields. At a sociological level, the fourth wave reflects an increasingly globalised crisis, with patterns of fentanyl use extending beyond North America to Europe and Asia [63]. Drug trafficking networks have adapted their strategies to distribute fentanyl and its analogues internationally, complicating control and prevention campaigns [64]. This is reflected in increased research efforts to develop regulatory control measures, considering the problem as an epidemic that needs to be taken into account in terms of age and gender factors in the affected population (“epidemic”, “gender factors”, “age distribution”). In addition, the persistence of stigmatisation and punitive policies towards people who use drugs remains a major obstacle to access to effective treatment and harm reduction.

On the other hand, interest in the use of fentanyl as an analgesic and anaesthetic, together with other narcotic and/or sedative substances such as benzodiazepines, has remained high throughout the four waves of opiate-related deaths, justified by its mechanism of action [65]. Beyond the historical and terminological analysis, the results of this study reveal the urgent need for the scientific community to critically reflect on the role of scientific publications in shaping the discourse on fentanyl. A research agenda that considers not only biomedical aspects but also social impacts and public health strategies to curb this phenomenon is necessary.

### 4.5. Limitations

The papers retrieved in this study were from WoS and PubMed coverage; so other papers indexed in other databases may not have been included.

Inevitably, those papers that (i) have not yet been assigned specific MeSH terms due to the novelty of the topic they deal with could not be included in this study; (ii) due to the periodic updating of MeSH terms, some recent studies may not be fully indexed with the most recent terms; (iii) depending on the temporal scope of the MeSH terms applied, some older studies may not be included if they were not correctly indexed with the terms available at that time.

On the other hand, the study was conducted to analyse the MeSH branches (C, D, F, G, and N) for their relevance to health, including mental health, but there are other branches that may be of interest, such as analytical, diagnostic, and therapeutic techniques, and equipment-related terms in the MeSH.

## 5. Conclusions

The evolution of scientific production on fentanyl in these five areas of OOC-related research shows that the focus has shifted from describing its pharmacological properties to a more holistic understanding that encompasses its social and behavioural effects. The intersection of these fields highlights the importance of addressing the opioid crisis. To effectively combat addictions and the current fentanyl crisis, it is necessary to integrate advances in different areas of knowledge through convergence and interdisciplinary research. To generate more effective responses to the growing impact of fentanyl use and dependence, transdisciplinary research integrating clinical, social, and political perspectives on fentanyl must be promoted. This approach encourages the scientific community to adopt a more comprehensive perspective within their own disciplines, connecting their findings to the real-life experiences of patients and individuals who use fentanyl illegally. This will inform policies and knowledge on this issue.

## Data Availability

The datasets generated and/or analysed during the current study are available in the Zenodo repository. Available from: https://doi.org/10.5281/zenodo.13141576.

## Supplementary Materials

The following supporting information can be downloaded at: https://www.mdpi.com/article/10.3390/jcm14155187/s1, Supplementary File S1. Search equation for publications on Opioids.

## References

[1] Makary M.A., Overton H.N., Wang P. (2017). Overprescribing Is Major Contributor to Opioid Crisis. BMJ.

[2] National Institute on Drug Abuse (NIDA) Drug Overdose Deaths: Facts and Figures. https://nida.nih.gov/research-topics/trends-statistics/overdose-death-rates.

[3] Gardner E.A., McGrath S.A., Dowling D., Bai D. (2022). The Opioid Crisis: Prevalence and Markets of Opioids. Forensic Sci. Rev..

[4] Schieber L.Z., Guy G.P., Seth P., Losby J.L. (2020). Variation in Adult Outpatient Opioid Prescription Dispensing by Age and Sex—United States, 2008–2018. MMWR Morb. Mortal. Wkly. Rep..

[5] Bloschichak A., Hargraves J. Opioid Prescriptions Declined 32% for the Commercially Insured over 10 Years (2008 to 2017). https://healthcostinstitute.org/hcci-originals-dropdown/all-hcci-reports/opioid-10yr-trends.

[6] Gisev N., Pearson S.-A., Larance B., Larney S., Blanch B., Degenhardt L. (2019). A Population-Based Study of Transdermal Fentanyl Initiation in Australian Clinical Practice. Eur. J. Clin. Pharmacol..

[7] Mossey J.M. (2011). Defining Racial and Ethnic Disparities in Pain Management. Clin. Orthop..

[8] Dowell D., Ragan K.R., Jones C.M., Baldwin G.T., Chou R. (2022). CDC Clinical Practice Guideline for Prescribing Opioids for Pain—United States, 2022. MMWR Recomm. Rep. Morb. Mortal. Wkly. Rep. Recomm. Rep..

[9] Pletcher M.J., Kertesz S.G., Kohn M.A., Gonzales R. (2008). Trends in Opioid Prescribing by Race/Ethnicity for Patients Seeking Care in US Emergency Departments. JAMA.

[10] Dahlhamer J.M., Connor E.M., Bose J., Lucas J.L., Zelaya C.E. (2021). Prescription Opioid Use Among Adults with Chronic Pain: United States, 2019. Natl. Health Stat. Rep..

[11] Manchikanti L., Singh V.M., Staats P.S., Trescot A.M., Prunskis J., Knezevic N.N., Soin A., Kaye A.D., Atluri S., Boswell M.V. (2022). Fourth Wave of Opioid (Illicit Drug) Overdose Deaths and Diminishing Access to Prescription Opioids and Interventional Techniques: Cause and Effect. Pain. Physician.

[12] Friedman J., Montero F., Bourgois P., Wahbi R., Dye D., Goodman-Meza D., Shover C. (2022). Xylazine Spreads across the US: A Growing Component of the Increasingly Synthetic and Polysubstance Overdose Crisis. Drug Alcohol. Depend..

[13] Estadt A.T., White B.N., Ricks J.M., Lancaster K.E., Hepler S., Miller W.C., Kline D. (2024). The Impact of Fentanyl on State-and County-Level Psychostimulant and Cocaine Overdose Death Rates by Race in Ohio from 2010 to 2020: A Time Series and Spatiotemporal Analysis. Harm. Reduct. J..

[14] Kline D., Bunting A.M., Hepler S.A., Rivera-Aguirre A., Krawczyk N., Cerda M. (2023). State-Level History of Overdose Deaths Involving Stimulants in the United States, 1999–2020. Am. J. Public. Health.

[15] Zoorob M. (2019). Fentanyl Shock: The Changing Geography of Overdose in the United States. Int. J. Drug Policy.

[16] Sauer J., Stewart K. (2023). Geographic Information Science and the United States Opioid Overdose Crisis: A Scoping Review of Methods, Scales, and Application Areas. Soc. Sci. Med..

[17] Mattson C.L., Tanz L.J., Quinn K., Kariisa M., Patel P., Davis N.L. (2021). Trends and Geographic Patterns in Drug and Synthetic Opioid Overdose Deaths—United States, 2013–2019. MMWR Morb. Mortal. Wkly. Rep..

[18] Ciccarone D. (2021). The Rise of Illicit Fentanyls, Stimulants and the Fourth Wave of the Opioid Overdose Crisis. Curr. Opin. Psychiatry.

[19] Adinolfi I. Parliamentary Question|Europe’s Escalating Fentanyl Crisis|E-003085/2023|European Parliament. https://www.europarl.europa.eu/doceo/document/E-9-2023-003085_EN.html.

[20] Moncrieff T., Moncrieff J. (2023). A Comparison of Opioid Prescription Trends in England and the United States from 2008 to 2020. Int. J. Risk Saf. Med..

[21] Kalkman G.A., Kramers C., van den Brink W., Schellekens A.F.A. (2022). The North American Opioid Crisis: A European Perspective. Lancet Lond. Engl..

[22] Bosetti C., Santucci C., Radrezza S., Erthal J., Berterame S., Corli O. (2019). Trends in the Consumption of Opioids for the Treatment of Severe Pain in Europe, 1990–2016. Eur. J. Pain. Lond. Engl..

[23] Lei F., Ye J., Wang J., Xia Z. (2019). A Bibliometric Analysis of Publications on Oxycodone from 1998 to 2017. BioMed Res. Int..

[24] Akbar H.F., Siddiq K., Nusrat S. (2019). Citation Classics and Trends in the Field of Opioids: A Bibliometric Analysis. Cureus.

[25] Sixto-Costoya A., García-Zorita C., Valderrama-Zurián J.C., Sanz-Casado E., Serrano-López A.E. (2025). Evolution of Marijuana Research at the Biopsychosocial Level: A General View. Int. J. Ment. Health Addict..

[26] Valderrama-Zurián J.C., García-Zorita C., Marugán-Lázaro S., Sanz-Casado E. (2021). Comparison of MeSH Terms and KeyWords Plus Terms for More Accurate Classification in Medical Research Fields. A Case Study in Cannabis Research. Inf. Process. Manag..

[27] R Core Team R: A Language and Environment for Statistical Computing. https://joaquimllisterri.cat/phonetics/fon_R/R.html.

[28] Lê S., Josse J., Husson F. (2008). FactoMineR: An R Package for Multivariate Analysis. J. Stat. Softw..

[29] Greenacre M. (2007). Correspondence Analysis in Practice.

[30] Manchikanti L., Helm S., Fellows B., Janata J.W., Pampati V., Grider J.S., Boswell M.V. (2012). Opioid Epidemic in the United States. Pain. Physician.

[31] Kiyatkin E.A. (2019). Respiratory Depression and Brain Hypoxia Induced by Opioid Drugs: Morphine, Oxycodone, Heroin, and Fentanyl. Neuropharmacology.

[32] Kibaly C., Alderete J.A., Liu S.H., Nasef H.S., Law P.-Y., Evans C.J., Cahill C.M. (2021). Oxycodone in the Opioid Epidemic: High “Liking”, “Wanting”, and Abuse Liability. Cell. Mol. Neurobiol..

[33] Busse J.W., Craigie S., Juurlink D.N., Buckley D.N., Wang L., Couban R.J., Agoritsas T., Akl E.A., Carrasco-Labra A., Cooper L. (2017). Guideline for Opioid Therapy and Chronic Noncancer Pain. J. Assoc. Medicale Can..

[34] (2016). CDC Guideline for Prescribing Opioids for Chronic Pain—United States, 2016. MMWR Recomm. Rep..

[35] Jobski K., Bantel C., Hoffmann F. (2023). Abuse, Dependence and Withdrawal Associated with Fentanyl and the Role of Its (Designated) Route of Administration: An Analysis of Spontaneous Reports from Europe. Eur. J. Clin. Pharmacol..

[36] Tournebize J., Gibaja V., Frauger E., Authier N., Seyer D., Perri-Plandé J., Fresse A., Gillet P., Javot L., Kahn J.-P. (2020). French trends in the misuse of Fentanyl: From 2010 to 2015. Therapie.

[37] Mercadante S., Prestia G., Adile C., Casuccio A. (2014). Intranasal Fentanyl Versus Fentanyl Pectin Nasal Spray for the Management of Breakthrough Cancer Pain in Doses Proportional to Basal Opioid Regimen. J. Pain..

[38] Frenk S.M., Porter K.S., Paulozzi L. (2015). Prescription Opioid Analgesic Use Among Adults: United States, 1999–2012.

[39] Preuss C.V., Kalava A., King K.C. (2024). Prescription of Controlled Substances: Benefits and Risks. StatPearls.

[40] Muijsers R.B., Wagstaff A.J. (2001). Transdermal Fentanyl: An Updated Review of Its Pharmacological Properties and Therapeutic Efficacy in Chronic Cancer Pain Control. Drugs.

[41] Halliburton J.R. (1988). The Pharmacokinetics of Fentanyl, Sufentanil and Alfentanil: A Comparative Review. AANA J..

[42] Maguire P., Tsai N., Kamal J., Cometta-Morini C., Upton C., Loew G. (1992). Pharmacological Profiles of Fentanyl Analogs at Mu, Delta and Kappa Opiate Receptors. Eur. J. Pharmacol..

[43] Calderón E., Pernia A., Román M.D., Pérez A.C., Torres L.M. (2003). Analgesia and sedation in the subarachnoid anesthesia technique: Comparative study between remifentanil and fentanyl/midazolam. Rev. Esp. Anestesiol. Reanim..

[44] Soysal S., Karcioglu O., Demircan A., Topacoglu H., Serinken M., Ozucelik N., Tirpan K., Gunerli A. (2004). Comparison of Meperidine plus Midazolam and Fentanyl plus Midazolam in Procedural Sedation: A Double-Blind, Randomized Controlled Trial. Adv. Ther..

[45] Dickson-Gomez J., Krechel S., Spector A., Weeks M., Ohlrich J., Green Montaque H.D., Li J. (2022). The Effects of Opioid Policy Changes on Transitions from Prescription Opioids to Heroin, Fentanyl and Injection Drug Use: A Qualitative Analysis. Subst. Abuse Treat. Prev. Policy.

[46] Salani D., Mckay M., Zdanowicz M. (2020). The Deadly Trio: Heroin, FentaNYL, and Carfentanil. J. Emerg. Nurs..

[47] Lambdin B.H., Bluthenthal R.N., Wenger L.D., Wheeler E., Garner B., Lakosky P., Kral A.H. (2020). Overdose Education and Naloxone Distribution Within Syringe Service Programs—United States, 2019. MMWR Morb. Mortal. Wkly. Rep..

[48] Cone K., Lanpher J., Kinens A., Richard P., Couture S., Brackin R., Payne E., Harrington K., Rice K.C., Stevenson G.W. (2018). Delta/Mu Opioid Receptor Interactions in Operant Conditioning Assays of Pain-Depressed Responding and Drug-Induced Rate Suppression: Assessment of Therapeutic Index in Male Sprague Dawley Rats. Psychopharmacology.

[49] Cooper Z.D., Shi Y.-G., Woods J.H. (2010). Reinforcer-Dependent Enhancement of Operant Responding in Opioid-Withdrawn Rats. Psychopharmacology.

[50] Bozarth M.A. (1994). Opiate Reinforcement Processes: Re-Assembling Multiple Mechanisms. Addict. Abingdon Engl..

[51] Sigmon S.C., Bisaga A., Nunes E.V., O’Connor P.G., Kosten T., Woody G. (2012). Opioid Detoxification and Naltrexone Induction Strategies: Recommendations for Clinical Practice. Am. J. Drug Alcohol. Abuse.

[52] Armenian P., Vo K.T., Barr-Walker J., Lynch K.L. (2018). Fentanyl, Fentanyl Analogs and Novel Synthetic Opioids: A Comprehensive Review. Neuropharmacology.

[53] National Institute on Drug Abuse (NIDA) Drugs, Brains, and Behavior: The Science of Addiction—Preface. https://nida.nih.gov/research-topics/addiction-science/drugs-brain-behavior-science-of-addiction.

[54] van Draanen J., Tsang C., Mitra S., Phuong V., Murakami A., Karamouzian M., Richardson L. (2022). Mental Disorder and Opioid Overdose: A Systematic Review. Soc. Psychiatry Psychiatr. Epidemiol..

[55] Downs A., Walter L., Shelton R., Li L. (2025). Co-Occurring Illicit Fentanyl Use and Psychiatric Disorders in Emergency Department Patients. Sci. Rep..

[56] Wagner K.D., Fiuty P., Page K., Tracy E.C., Nocera M., Miller C.W., Tarhuni L.J., Dasgupta N. (2023). Prevalence of Fentanyl in Methamphetamine and Cocaine Samples Collected by Community-Based Drug Checking Services. Drug Alcohol. Depend..

[57] Guo Y., Yu R., Zhang T., Ren F., Yu Z., Cheng J., Jia H., Shi W., Zhang Y. (2024). Synthesis and Biological Evaluation of Novel Biased Mu-Opioid Receptor Agonists. Molecules.

[58] Pagare P.P., Li M., Zheng Y., Kulkarni A.S., Obeng S., Huang B., Ruiz C., Gillespie J.C., Mendez R.E., Stevens D.L. (2022). Design, Synthesis, and Biological Evaluation of NAP Isosteres: A Switch from Peripheral to Central Nervous System Acting Mu-Opioid Receptor Antagonists. J. Med. Chem..

[59] Rudd R.A., Seth P., David F., Scholl L. (2016). Increases in Drug and Opioid-Involved Overdose Deaths—United States, 2010–2015. MMWR Morb. Mortal. Wkly. Rep..

[60] Machado-Vieira R., Zarate C.A. (2011). Proof of Concept Trials in Bipolar Disorder and Major Depressive Disorder: A Translational Perspective in the Search for Improved Treatments. Depress. Anxiety.

[61] Gerra G., Borella F., Zaimovic A., Moi G., Bussandri M., Bubici C., Bertacca S. (2004). Buprenorphine versus Methadone for Opioid Dependence: Predictor Variables for Treatment Outcome. Drug Alcohol. Depend..

[62] Sud A., Strang M., Buchman D.Z., Spithoff S., Upshur R.E.G., Webster F., Grundy Q. (2022). How the Suboxone Education Programme Presented as a Solution to Risks in the Canadian Opioid Crisis: A Critical Discourse Analysis. BMJ Open.

[63] European Union Drugs Agency EU Drug Market: Heroin and Other Opioids—Global Context. https://www.euda.europa.eu/publications/eu-drug-markets/heroin-and-other-opioids/global-context_en.

[64] U.S. Drug Enforcement Administration 2020 National Drug Threat Assessment. https://www.dea.gov/documents/2021/03/02/2020-national-drug-threat-assessment.

[65] Fonseca N.M., Guimarães G.M.N., Pontes J.P.J., de Araujo Azi L.M.T., de Ávila Oliveira R. (2023). Safety and Effectiveness of Adding Fentanyl or Sufentanil to Spinal Anesthesia: Systematic Review and Meta-Analysis of Randomized Controlled Trials. Braz. J. Anesthesiol. Engl. Ed..

