# Supplementary material for: Fentanyl Research: Key to Fighting the Opioid Crisis
Source: J Clin Med. 2025 Jul 22;14(15):5187. doi: 10.3390/jcm14155187 (PMC12348003; doi:10.3390/jcm14155187)
Supplement: Supplementary file 1 [file jcm-14-05187-s001.zip › jcm-3726960-supplementary.pdf]

Supplementary File S1. Search equation for publications on Opioids

The following search equation was used to obtain the international scientific production (articles and reviews) on opioids up to 2022. Subsequently, the Meso Topics indicated in this document were excluded for lack of relevance to the objective of the study, obtaining a total of 53,670 documents.

(1 OR 2 OR 3) AND (4 OR 5 OR 6) = 53,670 documents

1

TITLE= (“overdose” OR (“substance NEAR/4 use”) OR “abuse*” OR “misuse*” OR “addict*” OR “abstinence” OR (“drug NEAR/4 rehabilitation”) OR (“drug* NEAR/4 use*”) OR “dependence” OR “dependency” OR “dependenc*” OR (“related NEAR/4 disorder”) OR (“use NEAR/4 disorder*”) OR “craving” OR “withdrawal” OR “intoxication” OR (“drug NEAR/4 market”) OR (“street NEAR/4 market”))

2

AUTHOR KEYWORDS= (“overdose” OR (“substance NEAR/4 use”) OR “abuse*” OR “misuse*” OR “addict*” OR “abstinence” OR (“drug NEAR/4 rehabilitation”) OR (“drug* NEAR/4 use*”) OR “dependence” OR “dependency” OR “dependenc*” OR (“related NEAR/4 disorder”) OR (“use NEAR/4 disorder*”) OR “craving” OR “withdrawal” OR “intoxication” OR (“drug NEAR/4 market”) OR (“street NEAR/4 market”))

3

ABSTRACT= (“overdose” OR (“substance NEAR/4 use”) OR “abuse*” OR “misuse*” OR “addict*” OR “abstinence” OR (“drug NEAR/4 rehabilitation”) OR (“drug* NEAR/4 use*”) OR “dependence” OR “dependency” OR “dependenc*” OR (“related NEAR/4 disorder”) OR (“use NEAR/4 disorder*”) OR “craving” OR “withdrawal” OR “intoxication” OR (“drug NEAR/4 market”) OR (“street NEAR/4 market”))

4

(ABSTRACT= (“opioid*” OR “heroin*” OR “fentanyl” OR “oxycodone” OR “hydrocodone” OR “opiate*” OR “3-Methylfentanyl” OR “4-anilino-N-phenethylpiperidine” OR “4-Chloroisobutyrfentanyl” OR “4-Fluorofentanyl” OR “4-Fluoroisobutyrfentanyl” OR “4-Methoxybutyrfentanyl” OR “Abstral” OR “Acrylfentanyl” OR “Actiq” OR “Adolonta” OR “Alfenta” OR “Alfentanil” OR “AMIDONE” OR “Aminobutene” OR “APADAZ” OR “APHOMORPHINE” OR “Avinza” OR “ benzimidazole” OR “BELBUCA” OR “BREAKYL” OR “BRIXADI” OR “Brorphine” OR “BROMANYL” OR “BUPRENEX” OR “BUPRENORFINA” OR "Buprenorphine" OR “Butrans” OR “Butyrfentanyl” OR “BUVIDAL” OR “CARFENTANIL” OR “carfentanyl” OR “CO-CODAMOL” OR “CO-CODAPRIN” OR “codeine” OR “CODAMINE” OR “Contramal” OR “Conzip” OR “COMBUNOX”

OR “Cyclopropylfentanyl” OR “Demerol” OR “dihydrocodeine” OR “Dilaudid” OR “DEPODUR” OR “Dolophine” OR “Duragesic” OR “DURAMORPH” OR “Durogesic” OR “EMBEDA” OR “EFFENTORA” OR “Eltram” OR “EPTADONE” OR “ETAZENE” OR “ETODESNITAZENE” OR “etonitazene” OR “Etorphine” OR “Exalgo” OR “FENDIVIA” OR “FENTANEST” OR “Fentora” OR “FIORINAL” OR (“Fluoro isobutyryl fentanyl”) OR “Furanylfentanyl” OR “FURANYL-FENTANYL” OR “HIDROCODONE” OR “HIDROMORPHONE” OR “Hycet” OR “Hycodan” OR “hydrocodone” OR “Hydromet” OR “hydromorphone” OR “HYDROXYMORPHONE” OR “Hysingla” OR “HY-PHEN” OR “INDOL” OR “INFUMORPH” OR “Instanyl” OR “Isotonitazene” OR “Ionsys” OR “Ixprim” OR “Kadian” OR “KYNMOBI” OR “Lazanda” OR “Lorcet” OR “Lortab” OR “Matrifen” OR “meperidine” OR “METHADONE” OR “Methadose” OR “Methoxyacetylfentanyl” OR “MORFINA” OR “Morphine” OR “Morphabond” OR “MS Contin” OR “ nitazene” OR “Norco” OR “Norfentanyl” OR “N-Phenethyl-4-piperidone” OR “Nucynta” OR “Numorphan” OR “OCFENTANIL” OR “O-CODEINE” OR “Onsolis” OR “Opana” OR “Oramorph” OR “Oxaydo” OR “OXFENTANIL” OR “OXICODONA” OR “OXICODONE” OR “Oxycet” OR “OxyContin” OR “oxymorphone” OR “OXYNORM” OR “Palexia” OR “Palladone” OR “PAZITAL” OR “PECFENT” OR “PENTAZOCINE” OR “Percocet” OR “Percodan” OR “PETHIDINE” OR “PHENERGAN” OR “Piperidylthiambutene” OR “PROBUPHINE” OR “PSEUDOPHEDRINE” OR “QDOLO” OR “Rapifen” OR “REMIFENTANIL” OR “Remifentanyl” OR “ROXICODONE” OR “SEVREDOL” OR “SIXMO” OR “SUBLOCADE” OR “SUBOXONE” OR “SUBUTEX” OR “sufentanil” OR “tapentadol” OR “TARGIN” OR “TARGINIQ” OR “ TIONER” OR “thiambutene” OR “TRAMADEX” OR “TRAMADOL” OR “TRAMADOLOL” OR “TRAMADOLOR” OR “TRAMAL” OR “TRAMAZAC” OR “TRAMOL” OR (“TRAMP TAB”) OR “TRICARE” OR “TRICARE” OR “TRODON” OR “U-47700” OR “ULTIVA” OR “ULTRACET” OR “ULTRAM” OR “VALERYLFENTANYL” OR “VANTRELA” OR “VICODIN” OR “VICOPROFEN” OR “VITUZ” OR “WILDNIL” OR “XARTEMIS” OR “XTAMPZA” OR “ZUBSOLV” OR “ZYTRAM” OR “ZOHYDRO”)) NOT (DOCUMENT TYPES=("EDITORIAL MATERIAL" OR "BOOK CHAPTER" OR "LETTER" OR "PROCEEDINGS PAPER" OR "EARLY ACCESS" OR "MEETING ABSTRACT" OR "BOOK" OR "REPRINT" OR "BOOK REVIEW" OR "RETRACTED PUBLICATION" OR "CORRECTION" OR "PUBLICATION WITH EXPRESSION OF CONCERN" OR "WITHDRAWN PUBLICATION" OR "BIOGRAPHICAL ITEM" OR "DATA PAPER" OR "MEETING")))

5

(TITLE= (“opioid*” OR “heroin*” OR “fentanyl” OR “oxycodone” OR “hydrocodone” OR “opiate*” OR “3-Methylfentanyl” OR “4-anilino-N-phenethylpiperidine” OR “4-Chloroisobutyrfentanyl” OR “4-Fluorofentanyl” OR “4-Fluoroisobutyrfentanyl” OR “4-Methoxybutyrfentanyl” OR “Abstral” OR “Acrylfentanyl” OR “Actiq” OR “Adolonta” OR “Alfenta” OR “Alfentanil” OR “AMIDONE” OR “Aminobutene” OR “APADAZ” OR “APHOMORPHINE” OR “Avinza” OR “ benzimidazole” OR “BELBUCA” OR “BREAKYL” OR “BRIXADI” OR “Brorphine” OR “BROMANYL” OR “BUPRENEX” OR “BUPRENORFINA” OR "Buprenorphine" OR “Butrans” OR “Butyrfentanyl” OR “BUVIDAL” OR “CARFENTANIL” OR “carfentanyl” OR “CO-

CODAMOL” OR “CO-CODAPRIN” OR “codeine” OR “CODAMINE” OR “Contramal” OR “Conzip” OR “COMBUNOX” OR “Cyclopropylfentanyl” OR “Demerol” OR “dihydrocodeine” OR “Dilaudid” OR “DEPODUR” OR “Dolophine” OR “Duragesic” OR “DURAMORPH” OR “Durogesic” OR “EMBEDA” OR “EFFENTORA” OR “Eltram” OR “EPTADONE” OR “ETAZENE” OR “ETODESNITAZENE” OR “etonitazene” OR “Etorphine” OR “Exalgo” OR “FENDIVIA” OR “FENTANEST” OR “Fentora” OR “FIORINAL” OR (“Fluoro isobutyryl fentanyl”) OR “Furanylfentanyl” OR “FURANYL-FENTANYL” OR “HIDROCODONE” OR “HIDROMORPHONE” OR “Hycet” OR “Hycodan” OR “hydrocodone” OR “Hydromet” OR “hydromorphone” OR “HYDROXYMORPHONE” OR “Hysingla” OR “HY-PHEN” OR “INDOL” OR “INFUMORPH” OR “Instanyl” OR “Isotonitazene” OR “Ionsys” OR “Ixprim” OR “Kadian” OR “KYNMOBI” OR “Lazanda” OR “Lorcet” OR “Lortab” OR “Matrifen” OR “meperidine” OR “METHADONE” OR “Methadose” OR “Methoxyacetylfentanyl” OR “MORFINA” OR “Morphine” OR “Morphabond” OR “MS Contin” OR “ nitazene” OR “Norco” OR “Norfentanyl” OR “N-Phenethyl-4-piperidone” OR “Nucynta” OR “Numorphan” OR “OCFENTANIL” OR “O-CODEINE” OR “Onsolis” OR “Opana” OR “Oramorph” OR “Oxaydo” OR “OXFENTANIL” OR “OXICODONA” OR “OXICODONE” OR “Oxycet” OR “OxyContin” OR “oxymorphone” OR “OXYNORM” OR “Palexia” OR “Palladone” OR “PAZITAL” OR “PECFENT” OR “PENTAZOCINE” OR “Percocet” OR “Percodan” OR “PETHIDINE” OR “PHENERGAN” OR “Piperidylthiambutene” OR “PROBUPHINE” OR “PSEUDOPHEDRINE” OR “QDOLO” OR “Rapifen” OR “REMIFENTANIL” OR “Remifentanyl” OR “ROXICODONE” OR “SEVREDOL” OR “SIXMO” OR “SUBLOCADE” OR “SUBOXONE” OR “SUBUTEX” OR “sufentanil” OR “tapentadol” OR “TARGIN” OR “TARGINIQ” OR “ TIONER” OR “thiambutene” OR “TRAMADEX” OR “TRAMADOL” OR “TRAMADOLOL” OR “TRAMADOLOR” OR “TRAMAL” OR “TRAMAZAC” OR “TRAMOL” OR (“TRAMP TAB”) OR “TRICARE” OR “TRICARE” OR “TRODON” OR “U-47700” OR “ULTIVA” OR “ULTRACET” OR “ULTRAM” OR “VALERYLFENTANYL” OR “VANTRELA” OR “VICODIN” OR “VICOPROFEN” OR “VITUZ” OR “WILDNIL” OR “XARTEMIS” OR “XTAMPZA” OR “ZUBSOLV” OR “ZYTRAM” OR “ZOHYDRO”)) NOT (DOCUMENT TYPES=("MEETING ABSTRACT" OR "EDITORIAL MATERIAL" OR "LETTER" OR "CORRECTION" OR "PROCEEDINGS PAPER" OR "NEWS ITEM" OR "BOOK CHAPTER" OR "EARLY ACCESS" OR "BOOK REVIEW" OR "RETRACTION" OR "BOOK" OR "RETRACTED PUBLICATION" OR "DATA PAPER" OR "REPRINT" OR "POETRY" OR "RECORD REVIEW" OR "THEATER REVIEW" OR "ART EXHIBIT REVIEW" OR "FILM REVIEW" OR "BIOGRAPHICAL ITEM" OR "EXPRESSION OF CONCERN" OR "TV REVIEW RADIO REVIEW" OR "PUBLICATION WITH EXPRESSION OF CONCERN")))

6

(AUTHOR KEYWORDS= (“opioid*” OR “heroin*” OR “fentanyl” OR “oxycodone” OR “hydrocodone” OR “opiate*” OR “3-Methylfentanyl” OR “4-anilino-N-phenethylpiperidine” OR “4-Chloroisobutyrfentanyl” OR “4-Fluorofentanyl” OR “4-Fluoroisobutyrfentanyl” OR “4-Methoxybutyrfentanyl” OR “Abstral” OR “Acrylfentanyl” OR “Actiq” OR “Adolonta” OR “Alfenta” OR “Alfentanil” OR

“AMIDONE” OR “Aminobutene” OR “APADAZ” OR “APHOMORPHINE” OR “Avinza” OR “ benzimidazole” OR “BELBUCA” OR “BREAKYL” OR “BRIXADI” OR “Brorphine” OR “BROMANYL” OR “BUPRENEX” OR “BUPRENORFINA” OR "Buprenorphine" OR “Butrans” OR “Butyrfentanyl” OR “BUVIDAL” OR “CARFENTANIL” OR “carfentanyl” OR “CO-CODAMOL” OR “CO-CODAPRIN” OR “codeine” OR “CODAMINE” OR “Contramal” OR “Conzip” OR “COMBUNOX” OR “Cyclopropylfentanyl” OR “Demerol” OR “dihydrocodeine” OR “Dilaudid” OR “DEPODUR” OR “Dolophine” OR “Duragesic” OR “DURAMORPH” OR “Durogesic” OR “EMBEDA” OR “EFFENTORA” OR “Eltram” OR “EPTADONE” OR “ETAZENE” OR “ETODESNITAZENE” OR “etonitazene” OR “Etorphine” OR “Exalgo” OR “FENDIVIA” OR “FENTANEST” OR “Fentora” OR “FIORINAL” OR (“Fluoro isobutyryl fentanyl”) OR “Furanylfentanyl” OR “FURANYL-FENTANYL” OR “HIDROCODONE” OR “HIDROMORPHONE” OR “Hycet” OR “Hycodan” OR “hydrocodone” OR “Hydromet” OR “hydromorphone” OR “HYDROXYMORPHONE” OR “Hysingla” OR “HY-PHEN” OR “INDOL” OR “INFUMORPH” OR “Instanyl” OR “Isotonitazene” OR “Ionsys” OR “Ixprim” OR “Kadian” OR “KYNMOBI” OR “Lazanda” OR “Lorcet” OR “Lortab” OR “Matrifen” OR “meperidine” OR “METHADONE” OR “Methadose” OR “Methoxyacetylfentanyl” OR “MORFINA” OR “Morphine” OR “Morphabond” OR “MS Contin” OR “ nitazene” OR “Norco” OR “Norfentanyl” OR “N-Phenethyl-4-piperidone” OR “Nucynta” OR “Numorphan” OR “OCFENTANIL” OR “O-CODEINE” OR “Onsolis” OR “Opana” OR “Oramorph” OR “Oxaydo” OR “OXFENTANIL” OR “OXICODONA” OR “OXICODONE” OR “Oxycet” OR “OxyContin” OR “oxymorphone” OR “OXYNORM” OR “Palexia” OR “Palladone” OR “PAZITAL” OR “PECFENT” OR “PENTAZOCINE” OR “Percocet” OR “Percodan” OR “PETHIDINE” OR “PHENERGAN” OR “Piperidylthiambutene” OR “PROBUPHINE” OR “PSEUDOPHEDRINE” OR “QDOLO” OR “Rapifen” OR “REMIFENTANIL” OR “Remifentanyl” OR “ROXICODONE” OR “SEVREDOL” OR “SIXMO” OR “SUBLOCADE” OR “SUBOXONE” OR “SUBUTEX” OR “sufentanil” OR “tapentadol” OR “TARGIN” OR “TARGINIQ” OR “ TIONER” OR “thiambutene” OR “TRAMADEX” OR “TRAMADOL” OR “TRAMADOLOL” OR “TRAMADOLOR” OR “TRAMAL” OR “TRAMAZAC” OR “TRAMOL” OR (“TRAMP TAB”) OR “TRICARE” OR “TRICARE” OR “TRODON” OR “U-47700” OR “ULTIVA” OR “ULTRACET” OR “ULTRAM” OR “VALERYLFENTANYL” OR “VANTRELA” OR “VICODIN” OR “VICOPROFEN” OR “VITUZ” OR “WILDNIL” OR “XARTEMIS” OR “XTAMPZA” OR “ZUBSOLV” OR “ZYTRAM” OR “ZOHYDRO”)) NOT (DOCUMENT TYPES=("MEETING ABSTRACT" OR "EDITORIAL MATERIAL" OR "PROCEEDINGS PAPER" OR "EARLY ACCESS" OR "LETTER" OR "BOOK CHAPTER" OR "CORRECTION" OR "RETRACTED PUBLICATION" OR "DATA PAPER" OR "BOOK REVIEW" OR "PUBLICATION WITH EXPRESSION OF CONCERN" OR "REPRINT" OR "POETRY" OR "WITHDRAWN PUBLICATION")))

The following Meso Citation Topics were excluded due to lack of relevance:

- Animal Sensing
- Applied Statistics & Probability
- Archaeology
- Archaeometry
- Biophotonics & Electromagnetic Field Safety
- Bioengineering
- Catalysts
- Chemometrics
- Climate Change
- Computer Vision & Graphics
- Crop Science
- Dairy & Animal Sciences
- Dynamical Systems & Time Dependence
- Economics
- Electrochemistry
- Entomology
- Explosives
- Herbicides, Pesticides & Ground Poisoning
- History & Philosophy Of Science
- History Of Medicine
- Ionic, Molecular & Complex Liquids
- Knowledge Engineering & Representation
- Laser Science
- Literary Theory
- Marine Biology
- Mass Spectrometry
- Membrane Science

- Microfluidic Devices & Superhydrophobicity
- Modern History
- Nanofibers, Scaffolds & Fabrication
- Nanoparticles
- Nitroxides, Antioxidants & Free Radicals
- Numerical Methods
- Ocean Dynamics
- Oceanography, Meteorology & Atmospheric Sciences
- Optical Chemistry
- Organic Semiconductors
- Philosophy
- Physical Chemistry
- Photocatalysts
- Photochemistry
- Photoluminescence
- Photoproductivity
- Phytochemicals
- Plant Pathology
- Political Philosophy
- Polymer Science
- Polymers & Macromolecules
- Radioactive Tracers
- Silicon Systems
- Soil Science
- Soviet, Russian & East European History
- Supply Chain & Logistics
- Surfactants, Lipid Bilayers & Antimicrobial Peptides
- Telecommunications
- Theater
- Thermodynamics

- Veterinary Sciences
- Water Treatment
- Wireless Technology
- Zoology & Animal Ecology
